# Does *Cathaya argyrophylla*, an ancient and threatened *Pinaceae* species endemic to China, show eco-physiological outliers to its *Pinaceae* relatives?

**DOI:** 10.1093/conphys/coaa094

**Published:** 2020-10-14

**Authors:** Dayong Fan, Xiangping Wang, Wangfeng Zhang, Xiangying Zhang, Shouren Zhang, Zongqiang Xie

**Affiliations:** 1 College of Forestry, Beijing Forestry University, 35 Qinghua East Road, Haidian District, Beijing 100083, China; 2 The Key Laboratory of Oasis Eco-Agriculture, Xinjiang Production and Construction Corps, Shihezi University, No. 221, Beisi Road, Shihezi 832000, China; 3 State Key Laboratory of Vegetation and Environmental Change, Institute of Botany, Chinese Academy of Sciences, No. 20, Xiangshan Nanxin Cun, Haidian District, Beijing 100093, China

**Keywords:** Hydraulic safety, photosynthetic capacity, phylogenetic signal, species’ distribution, polygenetic quantile regression

## Abstract

*Cathaya argyrophylla* is an ancient and threatened Pinaceae species endemic to China, but its eco-physiological traits are rarely reported. We hypothesized that *Cathaya* showed eco-physiological outliers to its Pinaceae relatives, which lead to its current endangered status. Here we collected the photosynthetic capacity (*P*_n_, maximum photosynthesis rate) and branchlet hydraulic safety (*P*_50_, the water potential at which a 50% loss in conductivity occurs) of Pinaceae species globally, including our measurements on *Cathaya*. We applied the phylogenetic comparative methods to investigate: (i) the phylogenetic signal of the two key traits across Pinaceae species, and (ii) the trait–climate relationships and the photosynthesis–cavitation resistance relationship across Pinaceae species. We applied the polygenetic quantile regression (PQR) method to assess whether *Cathaya* showed eco-physiological outliers to its Pinaceae relatives in terms of cavitation resistance and photosynthetic capacity. It was found that *P*_50_, and to a less extent, *P*_n_, had a strong phylogenetic signal consistent with niche conservation among Pinaceae species. Hydraulic safety largely determined non-threatened Pinaceae species’ distribution across moisture gradients at the global scale. There was also an adaptive trade-off relationship between *P*_n_ and *P*_50_. *Cathaya* is a high cavitation resistant, low photosynthetic capacity species. It showed eco-physiological outliers to its Pinaceae relatives because it had lower *P*_50_ and *P*_n_ below the 10% quantile boundaries along moisture and/or temperature gradients; also, it was above the 90% quantile boundary of the *P*_n_ and *P*_50_ relationship across non-endangered Pinaceae species. The PQR output demonstrated that in the subtropical area of China characterized by abundant rainfall, *Cathaya* has extra high hydraulic safety, suggesting inefficiency of carbon economy associated with either competition or other life history strategies, which lead to its current endangered status.

## Introduction

The ‘giant panda’ of the plant kingdom, *Cathaya argyrophylla* Chun et Kuang, was formally described in 1958 by Chun and Kuang (Chun, 1958; Ying *et al.*, 1983). It is a monotypic genus of *Pinaceae* endemic to China and has been categorized as a paleoendemic species, with a fossil history dating at least to the Cretaceous (Ferguson *et al.*, 1997; Liu *et al.*, 2000). *Cathaya* is one of the most vulnerable conifers in the world with total number of mature individuals less than 5000 (Xie *et al.*, 1999b). Having its first occurrence in North America and being a formerly widespread Asiatic species, *Cathaya* has now become severely restricted to only three sites in subtropical mountains of China as a consequence of Late Tertiary climatic deterioration and Quaternary glaciation (Sup. Fig. 1) (Liu and Basinger, 2000). Extant *Cathaya* is found in a few mesothermal, physiological arid (high water deficit), infertile and exposed slopes in this area (Taggart *et al.*, 2009; Xie *et al.*, 1999a). It was also found that the abilities of competition and recolonization of this species are very low and that the habitats suitable for this species are undergoing rapid deterioration and fragmentation (Fu, 1992; Xie and Chen, 1999); as such, identification of abiotic and biotic factors contributing to its endangered status is vital for its conservation purposes.

How has *Cathaya* been vulnerable to extinction? Probably, such limited adaptability to regional climates and environments are due to its unique eco-physiological traits (Hunter Jr *et al.*, 2006; Lusk, 2008). A species’ uniqueness can be shown in three ways: (i) outlier to certain traits of its relatives (Hunter Jr and Gibbs, 2006); (ii) outlier to trait–environment relationships among its relatives (Newsome *et al.*, 2020); and (iii) outlier to trait–trait relationships among its relatives (Brodribb *et al.*, 2008). In the first case, blind, unpigmented, cave-dwelling invertebrates, fishes and amphibians are good examples of special characteristics in response to a rare type of habitat (Culver *et al.*, 2000). In the second case, a good example is vertebrate species near the lower boundary of the relationship between habitat ranges and body sizes were more often in higher extinction risk categories (Newsome *et al.*, 2020). In the third case, a good example is the tropic unique flat-leaved pine, *Pinus krempfii*, showing departure from the trade-off pattern between leaf hydraulic conductivity and mesophyll path length across pine species. This eco-physiological outlier probably is a key factor attributing to its limited adaptability and endangered status (Brodribb *et al.*, 2008).

Although there is a large body of knowledge on the palaeoclimate, palaeogeology, anatomy, ecology, population genetics and evolutionary history of this species (Fu, 1992; Hu *et al.*, 1984; Liu and Basinger, 2000; Qian *et al.*, 2016; Ran *et al.*, 2018; Xie *et al.*, 1999b), the eco-physiological traits of *Cathaya* are rarely reported (Fan *et al.*, 2005; Zhang *et al.*, 2005). In the present study, we chose two key functional traits to test whether *Cathaya* shows eco-physiological outliers to its Pinaceae relatives. One is the drought tolerance assessed by the water potential at which a 50% loss in hydraulic conductivity occurs [*P*_50_, MPa (Choat *et al.*, 2012)]; another is the competition capacity using the maximum photosynthetic capacity [*P*_n_, μ mol CO_2_ m^−2^ s^−1^, (Augusto *et al.*, 2014)] as a surrogate. Theoretically, low *P*_50_ (high drought tolerance) means high carbon investment on xylem (e.g. high wood density, small tracheid size). The synthesis of xylem tissue is costly [6.5 and 11.8 mmol glucose per g of cellulose and lignin, respectively (Lambers *et al.*, 1992)]; as a result, less carbon investment to leaves and inefficiency of carbon economy (lower *P*_n_) are expected (Augusto *et al.*, 2014; Pittermann *et al.*, 2012). We thereby hypothesize that there exists a trade-off between *P*_50_ and *P*_n_ among non-threatened Pinaceae species, which has already been observed for the Cupressaeae species (Pittermann *et al.*, 2012).

It is well known that moisture gradient largely determines the distribution of species with different drought tolerances (Choat *et al.*, 2018; Choat *et al.*, 2006; Willson *et al.*, 2006). In the subtropical area of China characterized by abundant rainfall, common species should evolve trait suits with low drought tolerance. Therefore, it is possible that the restricted range of *Cathaya* is due to its extra-high drought tolerance, which would deviate from the general relationship between species drought tolerance and regional rainfall/water deficit gradient. Further, the high drought tolerance of *Cathaya* possibly comes at a significant cost on competition capacity, if there exists a trade-off between *P*_50_ and *P*_n_ among non-threatened Pinaceae species. As such, low competition capacity would make *Cathaya* hard to overcome local species’ competitive advantages (ecological barrier) for its survival and dispersion. However, other possibilities cannot be excluded; for example, *Cathaya* could deviate from the *P*_50_*–P*_n_ trade-off. Such departure means the participation of some other life history trade-offs and/or the regulation of some unusual structure characteristics (Brodribb *et al*., 2008).

The supposed unusual suites of traits of *Cathaya*, either due to evolutionary conservatism (e.g. more ancient Pinaceae species have higher drought tolerance) or due to developmental constraints (e.g. the rarity of major mutations) (Brodribb *et al.*, 2008; Hunter Jr and Gibbs, 2006; Lusk, 2008; Wiens *et al.*, 2005), could be evaluated preliminarily by phylogenetic niche conservatism (PNC). PNC is the tendency of lineages to retain their niche-related traits through speciation events (Harvey *et al.*, 1991; Ricklefs, 2010). Although it is a pattern, not a process, the initial general test can lead to specific tests addressing the hypothesized drivers (Crisp *et al.*, 2012). Additionally, as the cross-species correlations may be biased by a lack of statistical independence among closely related species with shared evolutionary history, phylogenetic signals are also needed to be known *a priori*, in order to minimize the Type I error rates of non-phylogenetically corrected cross-species correlations (Ackerly, 2000; Blomberg *et al.*, 2003).

In the present study, we addressed three questions: to detect (i) the phylogenetic signal of *P*_50_ and *P*_n_ across Pinaceae species, based on a global database and our measurements of *Cathaya*; (ii) which environmental variables drive the distribution of *P*_50_ and *P*_n_ across common Pinaceae species and if there exists a trade-off between *P*_50_ and *P*_n_; and (iii) if *Cathaya* shows outliers to the general relationships among common Pinaceae species between *P*_50_/*P*_n_ and environmental variables and the *P*_50_  *vs. P*_n_ trade-off relationship.

## Materials and methods

Field surveys were carried out at Jiaopengliao, Zixing City, Hunan Province, South China, with a geographic location of 25 ° 56 ′ 54 ″ N, 113 ° 29 ′ 24 ″E and an altitude of 1250 m, on the southwest part of Bamian Mountain Reserve. The forest was a natural mixed stand of conifer and broad-leaved trees. Besides *Cathaya*, there were also *Castanopsis* and *Altingia* tree species in the main canopy storey. The climate is a subtropical monsoon climate area with four distinct seasons and mild climate; the meteorological data could be seen in Supplementary Table 1. The main soil type is mountain yellow–brown earth, with a total nitrogen content of only 0.0061% (Zhang *et al.*, 2005). A total of 26 sunlit branches from six mature trees were selected for cavitation resistance measurement, and leaves from one branch from the six mature trees were selected for photosynthesis measurement.

### Light-response curve measurement

The light-response curve was measured from 9:00 to 12:00 AM in July with an infrared gas analyzer (LI-6400 Li-Cor, Lincoln, NE, USA) performed using the autoprogram function. One-year-old leaves were chosen for measurements. We chose the sequence of desired light settings of 1500; 1200; 800, 500, 300, 100, 50, 30 and 0 μmol (photon) m^−2^ s^−1^, a minimum wait time of 120 s and a maximum wait time of 200 s, and matching the infrared gas analyzers for the 50-μmol(CO_2_) mol(air)^−1^ difference in the CO_2_ concentration between the sample and the reference, which allowed them to be matched before every change in irradiance. Leaf temperature was kept between 24 and 26°C, and CO_2_ partial pressure of and relative humidity in the leaf cuvette was 60–80% during measurements. Leaf mass ratio (LMA) measurements were then taken on *P*_n_-measured specimens in the laboratory. Ten to 15 fully expanded healthy leaves, not including petioles, were selected (Pérez-Harguindeguy *et al.*, 2013). The stomatal conductance (*g*_s_) and transpiration rate (*T*_r_) at saturating irradiance was also recorded.

The light-response curve can be fitted by a non-rectangular hyperbola (Lambers *et al.*, 2008):

(1) \begin{equation*} A=\frac{\phi I+{P}_n-\sqrt{\left\{{\left(\phi I+{P}_n\right)}^2-4\Theta \phi I{P}_n\right\}}}{2\Theta}-{R}_{\mathrm{d}} \end{equation*}

where *A* is the net photosynthetic rate under specific irradiance; *P*_n_ is the light-saturated net photosynthetic rate; *I* is the irradiance; $\phi$ is the quantum yield of assimilation, which is the initial slope for the light-response curve; $\Theta$ is the convexity of the curve; and *R*_d_ is the dark respiration rate. $\phi$ and *R*_d_ were calculated using linear regression analysis (*A* to irradiance <150 μmol m^−2^ s^−1^), then *P*_n_ and $\Theta$ were derived empirically by fitting (1) to light response data. The light compensation point (LCP) was the irradiance when *A* = 0, below which there is insufficient light to compensate for respiratory carbon loss.

### Cavitation vulnerability curve and tracheid diameter measurements

Three to five branchlets (40–70 cm long, 2–4 years old) near that used for the light-response measurement were cut from each sample tree before sunrise (before 8:00 AM) to measure the specific conductivity (*k*_s_; kg m^−1^ MPa^−1^ s^−1^), leaf specific conductivity (*k*_l_; kg m^−1^ MPa^−1^ s^−1^) and cavitation vulnerability curve (totally 26 samples). The branchlet was wrapped in moist paper towels immediately after being cut and transported to the laboratory. All the samples were re-cut to 22–24 cm underwater, and all the measurements were carried out in an air-conditioned laboratory (26°C). The maximum flow rate was measured under 8 kPa hydrostatic pressure after air emboli were flushed out by perfusion with 110 kPa distilled water (flowing through 0.2 μm filter) for 30 min. Measurements were initiated after ~ 2 min when the flow rate stabilized. The weight of the collected efflux was measured every 30 s with a precision balance (Sartorius, BP221S, Göttingen, Germany) to obtain the flow rate. *k*_l_ was calculated by dividing the maximum flow rate by the total leaf area distal to the measured segment and by the pressure gradient. The leaf area was determined using a WinFOLIA system (Regent Instruments, Quebec City, Canada). *k*_s_ was calculated by dividing the maximum flow rate by the segment’s cross-section sapwood area. The total acropetal-end cross-section area of the branch segment was determined from its maximum and minimum diameters. The area of the pith was determined from its dimensions measured under a dissecting microscope equipped with a stage micrometer and subtracted from the above acropetal-end cross-section area to determine the cross-section sapwood area. The Huber value was calculated as the total cross-section sapwood area per unit leaf area (Tyree *et al.*, 1988).

The vulnerability of xylem to cavitation was characterized using a vulnerability curve, which was measured using a cavitation pressure chamber (PMS Instrument, Corvallis, Oregon, USA) according to Sperry and Saliendra (1994). A branch segment was inserted into a collar and sealed with both ends protruding. Air was injected into the collar at a set pressure, which was maintained for 15 min and then slowly decreased to 0.1 MPa. The hydraulic conductivity was then re-measured at a higher pressure. This procedure was repeated until at least 85% loss of hydraulic conductivity was reached. The percentage loss of conductivity (PLC) following each pressurization was calculated as PLC = 100 × (*K*_h_–*K*_h*i*_)/*K*_h_, where *K*_h*i*_ is the hydraulic conductivity measured at pressure *i*. The vulnerability curve for each sample was fitted with an exponential sigmoidal equation (Pammenter *et al.*, 1998):

(2) \begin{equation*} \textrm{PLC}=\frac{100}{1+{e}^{a\left(\varPsi -b\right)}} \end{equation*}

where *Ψ* is the negative of the injection pressure and *a* and *b* are coefficients estimated using a non-linear regression model with gnls() function of R software (R Development Core Team, 2016). The coefficient *b* represents *P*_50_, *a* represents the steepness of vulnerability curve. Wood density (*W*) was measured on stem segments used in the measurement of vulnerability curves after the removal of pith and bark, and the fresh volume was measured by the Archimedes principle of water displacement. The dry mass was determined after drying at 104°C for 24 h. *W* is expressed as dry mass per unit of fresh volume (g cm^−3^).

Three to five samples out of those used for VC curve construction were used for the anatomical measurements. Micro thin sections (12 μm thick) were taken, following the procedure descried in Domec *et al*. (2010). Those sections were stained with Safranin O and mounted in resin for image analysis using ImageJ (Media Cybernetics, Silver Spring, MD). The mean hydraulic diameter [*d*_h_ = Σ*d*^5^/Σ*d*^4^ where *d* = individual tracheid diameter (Kolb *et al.*, 1999)] was estimated. The equivalent circle diameter was taken as tracheid diameter based on the lumen area measured (Wheeler *et al.*, 2005). For each sample on pit-cambium transects separately, three radial files of tracheids were randomly selected, and all lumen areas were measured.

### The global datasets, tree phylogenies and phylogenetic comparative analyses

We collected three datasets in the present study: *P*_50_ dataset, *P*_n_ dataset and *P*_50_  *vs.*  *d*_h_ dataset. The branchlet *P*_50_ dataset used in the present study can be accessed from the TRY Plant Traits Database (https://www.try-db.org/TryWeb/Home.php) (Choat *et al*., 2012). We found 35 Pinaceae species (in addition to *Cathaya*) from the database with definite locations on the maximally resolved seed plant tree (Zanne *et al.*, 2014). Based on the *P*_50_ dataset, we selected the same Pinaceae species for the *P*_n_ dataset from the TRY Plant Traits Database. *P*_n_ with the same (or slightly different) latitude and longitude of the *P*_50_ dataset, measured under growth temperature, ambient CO_2_ and saturating irradiance, with VPD (vapour pressure deficit) ranging from 0.8 to 1.5 kPa and/or relative humidity from 55 to 80%, of sunlit leaves from mature trees, were chosen out of more than 14 000 records. *P*_n_ values of four Pinaceae species (*Abies concolor*, *Cedrus deodara*, *Pinus caribaea*, *Pinus echinata*) could not be found based on the selection criteria, and we added another two Pinaceae species’ data (*Pinus krempfii*, *Pinus kesiya*) from Brodribb *et al.* (2008), resulting in 34 Pinaceae species in the *P*_n_ dataset (including *Cathaya*). In the *P*_50_ dataset, there were 18 Pinaceae species with *P*_n_ measured at the same location (or a slightly different location with an elevation difference of <300 m); this sub-dataset was analyzed for the relationship between *P*_50_ and *P*_n_. At last, we collected eight literatures (Davis *et al.*, 1999; Domec *et al*., 2010; Hacke and Jansen 2009; Linton *et al.*, 1998; Maherali *et al.*, 2006; Mayr *et al.*, 2006; Oliveras *et al.*, 2003; Sterck *et al.*, 2012), in which both *d*_h_ and *P*_50_ of 14 Pinaceae species have been measured simultaneously, to construct the *P*_50_  *vs*. *d*_h_ dataset. Six environmental variables [latitude, altitude, mean annual temperature (MAT), mean annual precipitation (MAP), mean temperature in the coldest month (MTCM) and water deficit (WD)] were selected for trait–environment analysis. Climate data (means over 1950–2000) were extracted from the WorldClim2 database with a spatial resolution of ca. 1 km (Fick *et al.*, 2017) based on the geographic coordinates of sampling sites.

We then constructed the phylogenies for our species with the program Phylomatic (Webb *et al.*, 2008), using a maximally resolved seed plant tree (Zanne *et al*., 2014). Phylogenies were constructed for the *P*_50_, *P*_n_, *P*_50_  *vs.*  *P*_n_ and *P*_50_  *vs.*  *d*_h_ dataset (Supplementary Figs. 2 and 3 for *P*_50_ and *P*_n_). Afterwards, multiple approaches were applied to evaluate phylogenetic signals in *P*_50_ and *P*_n_. The first approach is Pagel’s *λ* calculation (Freckleton *et al.*, 2002) (using the ‘Phytools’ package in R). Secondly, the phylogenetic signal representation curve (PSR) approach has been adopted (Felizola Diniz Filho *et al.*, 2012; Hawkins *et al.*, 2014). The PSR area, expressing deviations from Brownian motion across the curve, is the same as Blomberg’s *K* statistic and can reveal whether traits are evolving at a slower (negative) or faster (positive) rate than expected under Brownian motion in different parts of the phylogeny (Felizola Diniz Filho *et al*., 2012). PSR curves for *P*_50_ and *P*_n_ were calculated by using the “PVR” package in R. The third approach (phylogenetic generalized least squares) followed Hawkins *et al.* (2014), Kozak *et al.* (2010) and Wiens *et al.* (2010). Three models of evolution for *P*_n_ and *P*_50_ were applied: a white noise (WN) model of random variation; a Brownian motion (BM) model of gradual and continuous drift in trait; and an Ornstein–Uhlenbeck (OU) model of constrained evolution. OU-constrained evolution (stabilizing selection) was regarded as the sign of phylogenetic signal to a more stringent sense (Crisp and Cook, 2012). The log-likelihood of phylogenetic generalized least squares fitting of the three models was calculated. The “GEIGER” package in R was used to calculate and compare the fit of each model using a likelihood test; the Akaike information criterion (AIC) derived from model likelihoods was used to determine whether each trait fits an OU model better than either a BM or WN model.

**Table 1:** The branchlet’s hydraulic traits and leaf photosynthetic traits of Cathaya measured in the present study

**Branchlet’s hydraulic traits**		**Photosynthetic traits**	
***P***_**50**_ (MPa)	−5.64 ± 0.39	***P***_**n**_ (μmol m^−2^ s^−1^)	5.96 ± 0.41
***k***_**s**_ (kg m^−1^ MPa^−1^ s^−1^)	0.45 ± 0.13	***g***_**s**_ (mmol m^−2^ s^−1^)	72.48 ± 10.07
***k***_**l**_ (kg m^−1^ MPa^−1^ s^−1^)	9.73 ± 4.05 E^−5^	***T***_***r***_ (mol m^−2^ s^−1^)	0.67 ± 0.13
**Huber**	2.31 ± 0.35 E^−4^	***LCP*** (μmol m^−2^ s^−1^)	12.41 ± 1.71
***d*** (μm)	6.86 ± 0.23	***R***_**d**_ (μmol m^−2^ s^−1^)	0.53 ± 0.07
***d***_**h**_ (μm)	9.95 ± 0.45	***LMA*** (g m^−2^)	204 ± 21
***W*** (g cm^−3^)	0.55 ± 0.07		

***P***_**50**_: the water potential at which a 50% loss in conductivity occurs; ***k***_**s**_: specific conductivity; ***k***_**l**_: leaf-specific conductivity; Huber: Huber value; ***d***: individual tracheid diameter; ***d***_**h**_: mean hydraulic diameter; ***W***: wood density; ***P***_**n**_: light-saturated net photosynthetic rate; ***g***_**s**_: stomatal conductance; ***T***_***r***_: transpiration rate; ***LCP***: light compensation point; ***R***_**d**_: dark respiration rate; LMA: leaf mass ratio.

To investigate the relationship between *P*_50_/*P*_n_ and eight environmental variables (including the first two axes of principal component analysis, PC1 and PC2) among Pinaceae species, a phylogenetic generalized linear model (PGLS) with branch length transformations optimized for the maximum likelihood was applied, using the ‘caper’ package in R. In PGLS analysis, regression parameters are found by maximum likelihood (ML) and ‘weighted’ by the variance–covariance matrix that represents the phylogenetic relationships among the species. The relationship between *P*_n_ and *P*_50_, *d*_h_ and *P*_50_ were also explored by PGLS. Assume that the pressure that causes the cavitation of water inside xylem tracheids is inversely proportional to the mean of the maximum (per tracheid) size of pit pores (*d*_p_) (air-seeding hypothesis (Zimmermann, 1983)) *P*_50_ ∝1/*d*_p_, and *d*_h_ is linear to the size of its largest pore: *d*_h_∝*d*_p_ (Martínez-Vilalta *et al.*, 2002), resulting in *P*_50_ ∝1/*d*_h_. Therefore, for the *P*_50_  *vs.*  *d*_h_ relationship investigation, − *P*_50_ and *d*_h_ were log-transformed beforehand.

To investigate whether *Cathaya* shows eco-physiological outliers to its Pinaceae relatives, the polygenetic quantile regression (PQR) approach was applied. Since *P*_50_/*P*_n_ is likely determined by multiple unmeasured factors, and species with trait values outside the quantile regression lines are more likely to be threatened with extinction, it is useful to define an approximate upper/lower boundary for *P*_50_/*P*_n_ at a given variable, rather than focusing on means (Hacke *et al.*, 2017; Newsome *et al.*, 2020). To conduct PQR across non-threatened species, we followed the method by Newsome *et al.* (2020). We firstly fitted the non-phylogenetic quantile regression model and obtained residuals; secondly, we constructed a phylogenetic “distance” matrix from the phylogenetic tree (one minus the correlation matrix); thirdly, we calculated the phylogenetic autocovariate using the inverse distance squared weighted mean of residuals; then, the phylogenetic autocovariate was included in the quantile regression model (we set the auto-covariate to its average value, thus the boundaries that we show are for species with average values of the residual auto-covariate). We fitted quantile regression models for the 90% and 10% quantiles to describe the upper and lower bounds, respectively. The PQR was carried out by using the ‘ape’ and ‘quantreg’ packages in R (Koenker *et al.*, 2018).

Among the collected *Pinaceae* data, seven species were listed on the IUCN red list (IUCN, 2018) above the ‘Vulnerable’ category; they are *Abies pinsapo* (En, endangered), *Cathaya argyrophylla* (Vu, vulnerable), *Cedrus libani* (Vu), *Cedrus brevifolia* (Vu), *Pinus albicaulis* (En), *Pinus caribaea* (En) and *Pinus krempfii* (Vu).

## Results

**Figure 1:**
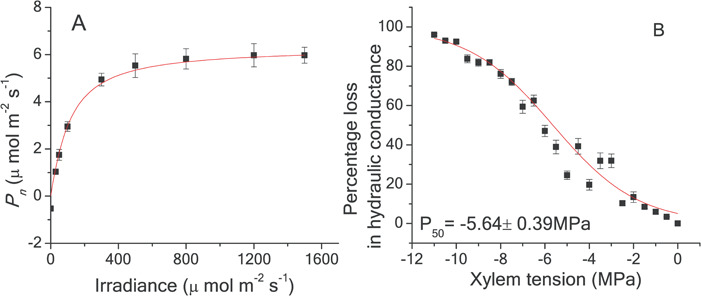
Light response curve (**A**) and percentage loss in hydraulic conductivity as a function of pressure for branchlets (**B**) of Cathaya. Curves were fitted using all data; only means (±1 SE) are presented. In subfigure A, the light-response curves were fitted by a nonrectangular hyperbola (*n* = 6). In subfigure B, the vulnerability curves were fitted with an exponential sigmoidal equation (*n* = 26)

The *P*_n_ and branchlet *P*_50_ of *Cathaya* were 5.96 μmol m^−2^ s^−1^ (Table 1; Fig. 1A) and −5.64 MPa (Table 1; Fig. 1B), respectively. *P*_n_ was in the lowest 25% quartile range among compiled Pinaceae species [Sup. Table 1, compiled *P*_n_ data of Pinaceae species can also be seen in Brodribb and Feild (2008)]. Besides two Cedrus species (*Cedrus libani*, *Cedrus brevifolia*), *Cathaya* was the most cavitation resistant one among compiled Pinaceae species (Sup. Table 1) [compiled *P*_50_ data of Pinaceae species can also be seen in Delucia *et al.* (2000) and Martínez-Vilalta *et al.* (2004)]. High resistance to cavitation indicated low hydraulic conductance (*k*_s_ and *k*_l_), as well as narrow tracheids (*d* and *d*_h_). In fact, the tracheid diameter of *Cathaya* was the narrowest one among the investigated Pinaceae species (Sup. Table 2; Sup. Fig. 3) [see also Hacke *et al*. (2017) and Martínez-Vilalta *et al.* (2012)]. The inefficiency of the carbon economy could also be indicated by low *g*_s_, *T*_r_ and *R*_d_ and high LMA (Table 1). The LCP (light compensation point) was 12.41 μmol m^−2^ s^−1^; however, we could not obtain a reliable LSP (light saturation point), as with the increase in irradiance, *P*_n_ increased slightly even at a very strong irradiance (>1500 μmol m^−2^ s^−1^); furthermore, there is no consensus on the accuracy of LSP estimation by a non-rectangular hyperbola (Li *et al*., 2019).

**Table 2:** Pagel’s *λ* and comparative fits of *P*_50_ and *P*_n_ to alternative evolutionary models of Pinaceae species, using phylogenetic generalized least squares

	Pagel’s λ		ΔAIC of model fit		
		*P*	BM	OU	WN
*P*_50_	0.992	<0.001	0.051	0	29.937
*P*_n_	0.526	0.063	5.511	0	1.154

Three models were applied, a Brownian motion (BM) model, an Ornstein–Uhlenbeck (OU) model and a White noise (WN) model. Akaike information criterion difference between model fits was used to evaluate which model is the best among those compared. Model with ΔAIC = 0 is the best evolutionary model for data fit. *P*: significance test between *λ* and the maximum likelihood value at *λ* = 0 (no phylogenetic dependence)

**Figure 2:**
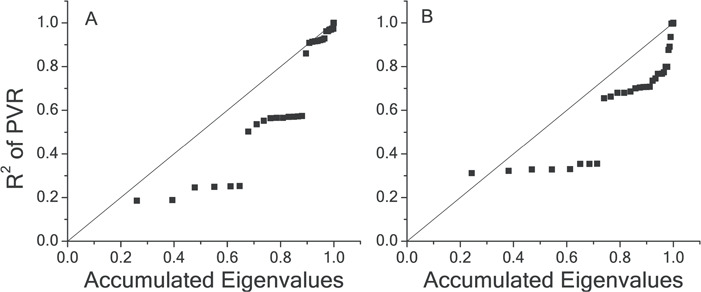
Phylogenetic signal representation curves (black squares) for (**A**) *P*_50_ and (**B**) *P*_n_ of Pinaceae species. The diagonal in the continuous traits is the relationship expected under a Brownian motion model of evolution

We calculated the phylogenetic signal of *P*_50_ and *P*_n_ based on global datasets of Pinaceae species; Pagel’s *λ* of *P*_50_ and Pn were 0.99 and 0.53, respectively (Table 2). *P*_50_ was different from the null hypothesis (no phylogenetic dependence), while *P*_n_ was marginally different from the null hypothesis, suggesting *P*_50_ was more phylogenetically conservative than *P*_n_. This could further be verified by the PSR area (Fig. 2); the PSR area for *P*_50_ was −0.16 while that for *P*_n_ was −0.11 (more negative area means more conservative). The fittings by phylogenetic generalized least squares showed that the Ornstein–Uhlenbeck (OU) model was the best model for comparative fits of *P*_50_ and *P*_n_ (Table 2), suggesting that both traits satisfied the most stringent definition of phylogenetic niche conservatism (Crisp and Cook, 2012; Hawkins *et al*., 2014; Losos, 2008).

Table 3 shows the PGLS slopes of *P*_50_ and *P*_n_ with eight environmental variables (including the first two axes of principal component analysis on the six environmental variables, i.e. PC1 and PC2). For the non-threatened species, *P*_50_ had significant associations with altitude, MAP, WD and PC2. There were no associations between *P*_n_ and environmental variables across all the common species, and only PC1 showed a marginal association with *P*_n_ (*P* = 0.091). PC1 was mostly represented by temperature variables (MAT and MTCM), while PC2 was mostly represented by moisture variables (water deficit index, negatively with MAP and positively with WD) (Table 3).

**Table 3:** Statistical significance [*P* value (determinant coefficient, *t* value)] of phylogenetic generalized linear model (PGLS) slopes between *P*_50_/*P*_n_ and eight environmental variables of non-threatened Pinaceae species

Traits	Latitude	Altitude	MAT	MAP	MTCM	WD	PC1	PC2
P_50_	0.59 (0.01,0.54)	**0.010** **(0.22,-2.78)**	0.561 (0.01,-0.59)	**0.013** **(0.21,2.64)**	0.714 (0.01,-0.37)	**0.052** **(0.13,-2.03)**	0.661 (0.01,-0.44)	**0.013** **(0.21,-2.65)**
Pn	0.322 (0.04,-1.02)	0.263 (0.05,-1.15)	0.181 (0.07,1.39)	0.189 (0.07,1.34)	0.262 (0.05,1.15)	0.661 (0.01,-0.44)	**0.091** **(0.11,1.70)**	0.793 (0.01,-0.27)
						
	*P*_50_	PC1	PC2	*P*_n_	PC1	PC2		
Latitude		−0.773	0.418		−0.762	0.077		
Altitude		−0.352	−0.446		−0.533	0.286		
MAT		0.916	0.359		0.99	0.014		
MAP		0.566	−0.776		0.185	−0.946		
MTCM		0.926	0.206		0.947	−0.047		
WD		0.080	0.863		0.491	0.85		

The first two axes of principal component analysis, PC1 and PC2, across all investigated species in *P*_50_ and *P*_n_ datasets were also demonstrated.

MAT: mean annual temperature (°C); MAP: mean annual precipitation (mm); MTCM: mean temperature in the coldest month (°C); WD: water deficit (mm).

Principal component analysis was conducted after the data have been Z-score standardized.

Numbers in bold mean significant relationships between *P*_50_/*P*_n_ and environmental variables of non-threatened Pinaceae species revealed by PGLS.

**Figure 3:**
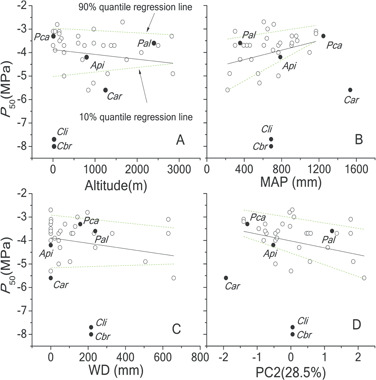
*P*_50_ as a function of (**A**) altitude; (**B**) MAP; (**C**) WD; and (**D**) PC2 (the second axis of principle component analysis on six environmental variables based on the *P*_50_ dataset, water deficit index) among non-threatened Pinaceae species globally. The black points showed the positions of endangered species. *Api*: *Abies pinsapo*. *Car*: *Cathaya argyrophylla*. *Cbr*: *Cedrus brevifolia*. *Cli*: *Cedrus libani*. *Pal*: *Pinus albicaulis*. *Pca*: *Pinus caribaea*. PC2 was largely represented by MAP (negatively) and WD (positively). The percentage in the bracket next to PC2 means how much of the variability in the data is accounted for by PC2. The phylogenetic generalized linear model (PGLS) with branch length transformations optimized for the maximum likelihood was applied. For PGLS models, Altitude: *P*_50_ = -4.05–0.17*Altitude (R^2^ = 0.22, t = −2.78, *P* = 0.011); MAP: *P*_50_ = -3.99 + 0.31*MAP (R^2^ = 0.21, t = 2.64, *P* = 0.013); WD: *P*_50_ = -4.05–0.22*WD (R^2^ = 0.13, t = −2.03, *P* = 0.052); PC2: *P*_50_ = -4.02–0.29*PC2 (R^2^ = 0.21, t = −2.65, *P* = 0.013). Principle component analysis (PCA) was conducted to extract the first two components (PC1 and PC2) from the six environmental *Z*-score standardized variables of all Pinaceae species investigated. Therefore, the PC1 and PC2 scores for endangered species have been obtained. The four PGLS models predicted the *P*_50_ of Cathaya should be −3.3~−4.2 MPa

**Figure 4:**
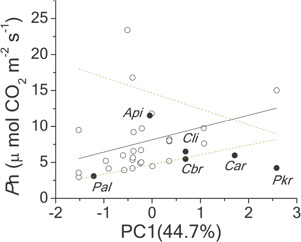
*P*_n_ as a function of PC1 among non-endangered Pinaceae species globally. The black points showed the positions of endangered species. *Api*: *Abies pinsapo*. *Car*: *Cathaya argyrophylla*, *Pkr*: *Pinus krempfii*, *Cbr*: *Cedrus brevifolia*, *Cli*: *Cedrus libani*. *Pal*: *Pinus albicaulis*. PC1 was best represented by MAT (positively) and MTCM (positively). The percentage in the bracket next to PC1 means how much of the variability in the data is accounted for by PC1. *Pkr* deviated from the global trend, in linear with Broddrib *et al*.’s (2008) observation. Among all PGLS outputs, only PC1 has a marginally significant correlation with *P*_n_ (*P* = 0.09). For PGLS models, *P*_n_ = 8.13 + 1.72*PC1 (R^2^ = 0.11, *t* = 1.70, *P* = 0.091)

Among non-threatened species, *P*_50_ was negatively related to altitude, WD and PC2 (water deficit index) and positively related to MAP (Fig. 3). If *Cathaya* and other five threatened species were positioned, *Cathaya* and two Cedrus (*Cedrus libani*, *Cedrus brevifolia*) showed significant deviations from the general trends: the three species had extraordinarily lower *P*_50_ than PGLS predictions and were below the 10% quantile boundaries. Further, the four PGLS models predicted that *P*_50_ of *Cathaya* should be −3.3~−4.2 MPa, while the measured value was −5.64 MPa. *P*_n_ was only related to PC1 (temperature index) (marginally, *P* = 0.091), indicating that higher photosynthetic capacity is associated with higher growth temperature (Fig. 4). Again *Cathaya* showed a sign of deviation from the general trend and was below the 10% quantile boundary. The PGLS model predicted that *P*_n_ should be around 11 μ mol CO_2_ m^−2^ s^−1^, while the measured value was 5.96 μmol m^−2^ s^−1^.

**Figure 5:**
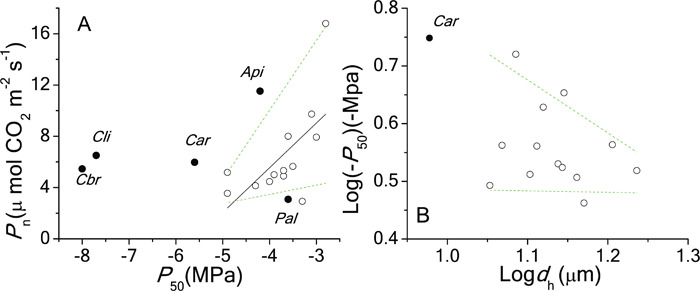
The trade-off relationships between *P*_n_ and *P*_50_ (**A**), and the mean hydraulic diameter (*d*_h_) and *P*_50_ (**B**) among no-threatened Pinaceae species globally. Phylogenetic generalized linear model (PGLS) with branch length transformations optimized for the maximum likelihood was applied. *Api: Abies pinsapo*, *Car: Cathaya argyrophylla*, *Cbr: Cedrus brevifolia*, *Cli: Cedrus libani*, *Pal: Pinus albicaulis*. The black points showed the positions of endangered species. For the PGLS model, *P*_n_ = 19.47 + 3.48*P_50_ (*R*^2^ = 0.21, *t* = −2.68, *P* = 0.022). There was no significant relationship between *d*_h_ and *P*_50_ (*R*^2^ = 0.03, *t* = −0.787, *P* = 0.44)

*P*_50_ was positively related to *P*_n_ (trade-off) (*P* = 0.02) among the 13 non-threatened Pinaceae species, when PGLS was applied (Fig. 5A). If *Cathaya* and other threatened species were positioned, *Cathaya* and the two Cedrus species deviated from the general trend and were above the 90% quantile boundary, which means they maintained considerable *P*_n_ at such low *P*_50_ levels. There existed no significant relationship between *d*_h_ and *P*_50_ among non-endangered species when PGLS was applied; despite that *Cathaya* had the narrowest tracheid, it was likely within the 10% and 90% quantile boundaries (Fig. 5B).

## Discussion

### Question 1: the phylogenetic signal of *P*_50_ and *P*_n_ across Pinaceae species

The hydraulic safety, which represents a realization of drought tolerance, had a strong phylogenetic signal consistent with niche conservation (Sup. Fig. 2A; Fig. 2A; Table 2). Moreover, to a less extent, the photosynthetic capacity, which represents a realization of carbon economy efficiency, also had a strong phylogenetic signal (Sup. Fig. 2B; Fig. 2B; Table 2). This could be validated by Pagel’s *λ*, PSR and model fitting comparison results. As such, not only morphological traits (Nobis *et al.*, 2012) but also the key physiological functions of hydraulic safety and photosynthetic capacity showed phylogenetic niche conservation (PNC) signals among Pinaceae species. On the other hand, Wang *et al.* (2018) found that *P*_n_ showed more PNC than *P*_50_ among nine *Picea* species in mid-China, which is opposite to our finding. This is probably due to the ‘local scale effect’ (Forrestel *et al.*, 2017) or statistical ‘funnel effect’ caused by the insufficient species amount investigated (Blomberg *et al*., 2003; Wright *et al.*, 2005). The strong PNC of *P*_50_ and/or *P*_n_ as revealed by the present study is consistent with the observation on other gymnosperms (Brodribb *et al.*, 2010; Pittermann *et al.*, 2010; Willson *et al.*, 2008). Ancient Pinaceae species tend to have high drought tolerance (low *P*_50_) and low photosynthetic capacity (low *P*_n_), in accordance with their initial diversification in the Permian, which was characterized by physiological aridity, and consequently they have a number of traits that provide physiological drought tolerance, permitting them to survive on frozen soils, deep sand and steep slopes (Graham, 1999).

### Question 2: the trait–environment relationship and the trade-off between *P*_50_ and *P*_n_

There existed significant PGLS relationships (slope) between altitude (negative), WD (negative) and *P*_50_ among non-threatened species. The results demonstrated that moisture gradient (WD and/or MAP) largely drives the distribution of species with different cavitation resistance (Table 3; Fig. 3). This could be further demonstrated from the significant PGLS negative slope of the second component (PC2) *vs.*  *P*_50_ relationship, while PC2 was largely determined by WD and MAP. High altitude, high WD and low MAP are conducive to high cavitation resistance (low *P*_50_) among non-threatened species. This output supports other studies that hydraulic safety largely determines species distribution across moisture gradients (Choat *et al.*, 2018; Choat *et al*., 2012; Niinemets and Valladares, 2006; Willson and Jackson, 2006), although most of which did not pay attention to phylogenetic dependence of inter-specific data. On the contrary, there existed very weak or no relationships between environmental variables and photosynthetic capacity among non-threatened species (Table 3), suggesting that hydraulic safety is more adaptive than photosynthetic capacity across Pinaceae species. Only temperature (PC1) executed a marginal impact on *P*_n_ (Fig. 4), which indicated the temperature dependence of enzymes’ kinetics responsible for carbon assimilation (Brooks *et al.*, 1985; Yamori *et al.*, 2005).

It has been demonstrated that the hydraulic efficiency of branch has a tight association with leaf photosynthetic capacity (Brodribb *et al.*, 2002; Scoffoni *et al.*, 2016; Xiong *et al.*, 2019). If there exists an adaptive trade-off relation between hydraulic efficiency and cavitation resistance among Pinaceae species (Choat *et al.*, 2011; Gleason *et al.*, 2016; Maherali *et al.*, 2004), it means photosynthesis could have a negative association with cavitation resistance among the non-threatened species globally. As expected by either the carbon economy theory (Augusto *et al*., 2014) or *k*_l_–*P*_n_ coordination (Brodribb *et al.*, 2002; Scoffoni *et al.*, 2016; Xiong *et al.*, 2019), the PGLS output of the 13 species did support the *P*_50_–*P*_n_ trade-off (Fig. 5A, *P* = 0.02), which was consistent with some other studies on gymnosperm species (Maherali *et al.*, 2006; Pittermann *et al*., 2012; Sterck *et al*., 2012). On the contrary, the non-significant association between *d*_h_ and *P*_50_ among the 14 Pinaceae species (Fig. 5B) did not support the theoretical model (Hacke *et al.*, 2004, Martínez-Vilalta *et al*., 2002; Mayr *et al*., 2006), which means that the torus–margo structure of gymnosperms (Choat *et al.*, 2009; Domec *et al.*, 2009; Hacke *et al.*, 2009) possibly masks the tracheid allometry deviated from the theoretical value based on relative simpler pit structure of angiosperms.

### Question 3: does *Cathaya* deviate from the general relationship among non-threatened Pinaceae species between *P*_50_/*P*_n_ and environmental variables?

*Cathaya* did show eco-physiological outliers of *P*_50_/*P*_n_ to its Pinaceae relatives. Besides two Cedrus species (*Cedrus libani*, *Cedrus brevifolia*), *Cathaya* was the most cavitation-resistant Pinaceae species among the Pinaceae species reported. *P*_n_ was in the lowest 25% quartile range among compiled Pinaceae species (Sup. Table 1). *Cathaya* also had the narrowest tracheid among the investigated Pinaceae species (Sup. Table 2; Fig. 5B). More importantly, *Cathaya* had *P*_50_ under the low (10%) quantile boundaries of *P*_50_  *vs*. environmental variables (altitude, MAP, WD and moisture index PC2) (Fig. 3) and *P*_n_ under the 10% quantile boundary of *P*_n_ along the temperature gradient (temperature index PC1, Fig. 4). The extra high hydraulic safety of *Cathaya* and low photosynthetic capacity are probably due to its ancientness (Sup. Fig. 2 A, B, Table 2, Fig. 2), not developmental constraint. Interestingly, unlike *Cathaya*, the extra high hydraulic safety of *Cedrus libani* and *Cedrus brevifolia* could not be explained by their recent evolutionary history and may largely be attributed by developmental constraint (Sup. Fig. 2 A, B). High cavitation resistance is associated with inefficiency of carbon economy, leading to *Cathaya*’s lower growth competition ability with its coexisting angiosperm species (Augusto *et al*., 2014; Brodribb *et al.*, 2007; Boyce *et al.*, 2009; Körner, 1995; Lusk *et al.*, 2003), and can only be confined to sites with arid (high water deficit), infertile and steep slopes in the subtropical mountains of South China.

However, it is worth noting that *Cathaya* was above the 90% quantile boundary of the *P*_n_ and *P*_50_ trade-off [although not as significant as the two Cedrus species (Fig. 5A)], indicating that it could maintain considerable *P*_n_ at such low *P*_50_ levels. The departure indicates the participation of some other life history trade-offs. For example, extra high cavitation resistance could come at a significant energy cost in terms of insufficient reproductive effort and does not necessarily compromise the competition capacity of mature individuals. Actually, it was found that the average seed weight of *Cathaya* was 0.0197 g [only 6% of the average seed weight of arbour species (Silvertown, 1982)], with over 60% of seeds without viability (Xie *et al.*, 2000), indicating insufficient energy input, although other mechanism(s) could not be overlooked (Hunter Jr and Gibbs, 2006; Vaario *et al.*, 2006; Wang *et al.*, 2006). The other mechanism(s) include specific arbuscular mycorrhizal root interaction (Vaario *et al.*, 2006) and low gene flow between isolated populations (Wang *et al.*, 2006). Another possibility is that some unusual structure characteristics such as torus thickness and depth of the pit chamber (Hacke and Jansen, 2009; Pittermann *et al.*, 2005) and tracheid wall reinforcement (Hacke *et al.*, 2017), but not tracheid diameter (Fig. 5B), drive the deviation. It is worth noting that *Cathaya* does not have tubular sclereids in the leaf (Dayong Fan, unpublished data), unlike the tropic unique flat-leaved pine, *Pinus krempfii* (Brodribb *et al.*, 2008). The torus-pit structure characteristics probably also led to the absence of the trade-off between *P*_50_ and *k*_s_ within *Cathaya* (Sup. Fig. 4A), but not the *P*_50_–wood density relationship (Sup. Fig. 4B). As such, the mechanism(s) underlying the deviation of *Cathaya* (and the two Cedrus species) from the *P*_n_ and *P*_50_ trade-off merits future investigation.

Interestingly, in the relationships between hydraulic safety and environment, three threatened species (*Pinus caribaea*, *Pinus albicaulis*, *Abies pinsapo*) were between the 10% and 90% quantile boundaries (Fig. 3), which were quite different from *Cathaya* and the two Cedrus species. The result suggests different mechanisms of endangerment for the six threatened species. For example, *Cathaya* and the two Cedrus were restricted to narrow range and habitats (Vidaković, 1991; Xie *et al.*, 1999b), since the site-specific environmental conditions might be quite different from the regional environmental conditions (the Climate data were extracted based on regional scale), and their deviations from trait–environment relationships could be expected, while for the other three species, they have broader distribution areas (Farjon, 1990; Thompson *et al.*, 1999), so probably other mechanisms [e.g. inter-specific interaction, human disturbance (Hunter Jr and Gibbs, 2006)] but not the limited adaptability (hydraulic safety) underpinned their vulnerability to extinction. As such, the PQR method on functional traits and their relationships to the environment can offer a useful tool to reveal the mechanism of species endangerment, in support of the viewpoint by Newsome *et al.* (2020). It is also worth noting that although the phylogenetic comparative methods applied in the present study could minimize the Type I error rates of non-phylogenetically corrected cross-species correlations (Ackerly, 2000; Blomberg *et al.*, 2003), other factors such as non-random sampling, intra-specific variation and measurement error could impact the output (Ives *et al.*, 2007; Huey *et al.*, 2019). As such, further investigation on the difference of eco-physiological traits between *Cathaya argyrophylla* and its Pinaceae relatives is warranted.

## Conclusions

In the present study, we found that the hydraulic safety, and to a less extent, photosynthetic capacity, had a strong phylogenetic signal consistent with niche conservation among Pinaceae species. Hydraulic safety largely determined the distribution of non-threatened Pinaceae species across moisture gradients at the global scale. There was a phylogenetic trade-off between hydraulic safety and photosynthesis across non-threatened Pinaceae species. *Cathaya* is a shade-intolerant, high cavitation resistant, low photosynthetic capacity Pinaceae species. These traits were largely attributed by its ancientness. *Cathaya* also showed eco-physiological outliers to its Pinaceae relatives because it had lower *P*_n_ and *P*_50_ outside the 10% quantile boundaries along moisture and/or temperature gradients. The safety–photosynthesis trade-off could provide an explanation on how *Cathaya* is vulnerable to extinction, but other factors contributing to its endangered status could not be overlooked. The somewhat departure of *Cathaya* from the safety–photosynthesis trade-off merits future investigation. We suggested that the PQR method could be a useful tool to reveal the mechanism of species endangerment of Pinaceae species under the global climate change.

## Supplementary Material

SupplementaryTable1_coaa094

SupplementaryTable2_coaa094

## References

[ref1] Ackerly DD (2000) Taxon sampling, correlated evolution, and independent contrasts. Evolution 54: 1480–1492. 11108577 10.1111/j.0014-3820.2000.tb00694.x

[ref2] Augusto L, Davies T, Delzon S, De Schrijver A (2014) The enigma of the rise of angiosperms: can we untie the knot? Ecol Lett 17: 1326–1338. 24975818 10.1111/ele.12323

[ref3] Blomberg SP, Garland T Jr, Ives AR (2003) Testing for phylogenetic signal in comparative data: behavioral traits are more labile. Evolution 57: 717–745. 12778543 10.1111/j.0014-3820.2003.tb00285.x

[ref4] Boyce CK, Brodribb TJ, Feild TS, Zwieniecki MA (2009) Angiosperm leaf vein evolution was physiologically and environmentally transformative. Philos Trans R Soc B Biol Sci 276: 1771–1776. 10.1098/rspb.2008.1919 PMC2674498 19324775

[ref5] Brodribb T, Holbrook NM, Gutierrez M (2002) Hydraulic and photosynthetic co-ordination in seasonally dry tropical forest trees. Plant Cell Environ 25: 1435–1444.

[ref6] Brodribb TJ, Feild TS (2008) Evolutionary significance of a flat-leaved pinus in vietnamese rainforest. New Phytol 178: 201–209. 18179604 10.1111/j.1469-8137.2007.02338.x

[ref7] Brodribb TJ, Feild TS (2010) Leaf hydraulic evolution led a surge in leaf photosynthetic capacity during early angiosperm diversification. Ecol Lett 13: 175–183. 19968696 10.1111/j.1461-0248.2009.01410.x

[ref8] Brodribb TJ, Feild TS, Jordan GJ (2007) Leaf maximum photosynthetic rate and venation are linked by hydraulics. Plant Physiol 144: 1890–1898. 17556506 10.1104/pp.107.101352 PMC1949879

[ref9] Brooks A, Farquhar G (1985) Effect of temperature on the co 2/o 2 specificity of ribulose-1, 5-bisphosphate carboxylase/oxygenase and the rate of respiration in the light. Planta 165: 397–406. 24241146 10.1007/BF00392238

[ref10] Choat B, Brodribb TJ, Brodersen CR, Duursma RA, Lopez R, Medlyn BE (2018) Triggers of tree mortality under drought. Nature 558: 531–539. 29950621 10.1038/s41586-018-0240-x

[ref11] Choat B et al. (2012) Global convergence in the vulnerability of forests to drought. Nature 491: 752–755. 23172141 10.1038/nature11688

[ref12] Choat B, Medek DE, Stuart SA, Pasquet-Kok J, Egerton JJ, Salari H, Sack L, Ball MC (2011) Xylem traits mediate a trade-off between resistance to freeze–thaw-induced embolism and photosynthetic capacity in overwintering evergreens. New Phytol 191: 996–1005. 21627664 10.1111/j.1469-8137.2011.03772.x

[ref13] Choat B, Pittermann J (2009) New insights into bordered pit structure and cavitation resistance in angiosperms and conifers. New Phytol 182: 557–560. 19422544 10.1111/j.1469-8137.2009.02847.x

[ref14] Chun W (1958) A new genus of pinaceae-cathaya chun et kuang gen. Nov., from the southern and western China. Botanicheskii Zhurnal SSSR 43: 461–470.

[ref15] Crisp MD, Cook LG (2012) Phylogenetic niche conservatism: what are the underlying evolutionary and ecological causes? New Phytol 196: 681–694. 22943495 10.1111/j.1469-8137.2012.04298.x

[ref16] Culver DC, Master LL, Christman MC, Hobbs HH III (2000) Obligate cave fauna of the 48 contiguous United States. Conserv Biol 14: 386–401.

[ref17] Davis SD, Sperry JS, Hacke UG (1999) The relationship between xylem conduit diameter and cavitation caused by freezing. Am J Bot 86: 1367–1372. 10523278

[ref18] Delucia EH, Maherali H, Carey EV (2000) Climate-driven changes in biomass allocation in pines. Global Change Biol 6: 587–593.

[ref19] Domec J-C, Schäfer K, Oren R, Kim HS, McCarthy HR (2010) Variable conductivity and embolism in roots and branches of four contrasting tree species and their impacts on whole-plant hydraulic performance under future atmospheric co2 concentration. Tree Physiol 30: 1001–1015. 20566583 10.1093/treephys/tpq054

[ref20] Domec J-C, Warren JM, Meinzer FC, Lachenbruch B (2009) Safety factors for xylem failure by implosion and air-seeding within roots, trunks and branches of young and old conifer trees. Iawa J 30: 101–120.

[ref21] Fan D, Zhang W, Chen Z, Xie Z (2005) Acclimation of *Cathaya argyrophylla* to light across a gradient of canopy openness. Acta Phytoecol Sinica 29: 713–723.

[ref22] Farjon A (1990) Pinaceae. Drawings and descriptions of the genera abies, cedrus, pseudolarix, keteleeria, nothotsuga, tsuga, cathaya, pseudotsuga, larix and picea. Koeltz scientific books.

[ref23] Felizola Diniz Filho JA, Rangel TF, Santos T, Mauricio Bini L (2012) Exploring patterns of interspecific variation in quantitative traits using sequential phylogenetic eigenvector regressions. Evolution 66: 1079–1090. 22486690 10.1111/j.1558-5646.2011.01499.x

[ref24] Ferguson D, Liu Y, Zetter R (1997) The Paleoendemic Plants of East Asia: Evidence From the Fossil Record for Changing Distribution Patterns. University of Hong Kong.

[ref25] Fick SE, Hijmans RJ (2017) Worldclim 2: new 1-km spatial resolution climate surfaces for global land areas. Int J Climatol 37: 4302–4315.

[ref26] Forrestel EJ, Donoghue MJ, Edwards EJ, Jetz W, Toit JC du, Smith MD (2017) Different clades and traits yield similar grassland functional responses. Proc Natl Acad Sci USA 114: 705–710. 28074042 10.1073/pnas.1612909114 PMC5278461

[ref27] Freckleton RP, Harvey PH, Pagel M (2002) Phylogenetic analysis and comparative data: a test and review of evidence. Am Nat 160: 712–726. 18707460 10.1086/343873

[ref28] Fu L (1992) The Red Book of Chinese Plants–Rare and Endangered Plants. Science Press, Beijing.

[ref29] Gleason SM et al. (2016) Weak tradeoff between xylem safety and xylem-specific hydraulic efficiency across the world’s woody plant species. New Phytol 209: 123–136. 26378984 10.1111/nph.13646

[ref30] Graham A (1999) Late Cretaceous and Cenozoic History of North American Vegetation: North of Mexico. Oxford University Press on Demand.

[ref31] Hacke UG, Jansen S (2009) Embolism resistance of three boreal conifer species varies with pit structure. New Phytol 182: 675–686. 19309447 10.1111/j.1469-8137.2009.02783.x

[ref32] Hacke UG, Sperry JS, Pittermann J (2004) Analysis of circular bordered pit function ii. Gymnosperm tracheids with torus-margo pit membranes. Am J Bot 91: 386–400. 21653394 10.3732/ajb.91.3.386

[ref33] Hacke UG, Spicer R, Schreiber SG, Plavcová L (2017) An ecophysiological and developmental perspective on variation in vessel diameter. Plant Cell Environ 40: 831–845. 27304704 10.1111/pce.12777

[ref34] Harvey PH, Pagel MD (1991) The Comparative Method in Evolutionary Biology. Oxford University Press Oxford.

[ref35] Hawkins BA, Rueda M, Rangel TF, Field R, Diniz-Filho JAF (2014) Community phylogenetics at the biogeographical scale: cold tolerance, niche conservatism and the structure of North American forests. J Biogeogr 41: 23–38. 24563577 10.1111/jbi.12171 PMC3920643

[ref36] Hu Y, Wang F (1984) Anatomical studies of Cathaya (Pinaceae). Am J Bot 71: 727–735.

[ref37] Huey RB, Garland T Jr, Turelli M (2019) Revisiting a key innovation in evolutionary biology: Felsenstein’s “phylogenies and the comparative method”. Am Nat 193: 755–772. 31094602 10.1086/703055

[ref38] Hunter ML Jr, Gibbs JP (2006) Fundamentals of Conservation Biology. John Wiley & Sons.

[ref39] IUCN (2018) The iucn red list of threatened species In Version 2018–1. Union for Conservation of Nature and Natural Resources, International.

[ref40] Ives AR, Midford PE, Garland T Jr (2007) Within-species variation and measurement error in phylogenetic comparative methods. Syst Biol 56: 252–270. 17464881 10.1080/10635150701313830

[ref41] Koenker R, Portnoy S, Ng P, Zeileis A, Grosjean P, Ripley B (2018) Quantreg: Quantile regression, version 5.3. 4. R package.

[ref42] Kolb KJ, Sperry JS (1999) Differences in drought adaptation between subspecies of sagebrush (*Artemisia tridentata*). Ecology 80: 2373–2384.

[ref43] Körner C (1995) Leaf diffusive conductances in the major vegetation types of the globe. Ecophysiology of Photosynthesis. Springer pp 463–490.

[ref44] Kozak KH, Wiens JJ (2010) Niche conservatism drives elevational diversity patterns in appalachian salamanders. Am Nat 176: 40–54. 20497055 10.1086/653031

[ref45] Lambers H, Chapin FS III, Pons TL (2008) Plant Physiological Ecology, Springer Science & Business Media.

[ref46] Lambers H, Poorter H (1992) Inherent variation in growth rate between higher plants: a search for physiological causes and ecological consequences. Advances in Ecological Research, Vol. 23. Elsevier, pp 187–261.

[ref47] Li YL, Liu XG, Hao K, Yang QL, Yang XQ, Zhang WH, Cong Y (2019) Light-response curve of photosynthesis and model fitting in leaves of *Mangifera indica* under different soil water conditions. Photosynthetica 57: 796–803.

[ref48] Linton M, Sperry J, Williams D (1998) Limits to water transport in *Juniperus osteosperma* and *Pinus edulis*: implications for drought tolerance and regulation of transpiration. Funct Ecol 12: 906–911.

[ref49] Liu Y, Basinger J (2000) Fossil Cathaya (Pinaceae) pollen from the Canadian High Arctic. Int J Plant Sci 161: 829–847.

[ref50] Losos JB (2008) Phylogenetic niche conservatism, phylogenetic signal and the relationship between phylogenetic relatedness and ecological similarity among species. Ecol Lett 11: 995–1003. 18673385 10.1111/j.1461-0248.2008.01229.x

[ref51] Lusk C (2008) Constraints on the evolution and geographical range of pinus. New Phytol 178: 1–3. 18315695 10.1111/j.1469-8137.2008.02371.x

[ref52] Lusk CH, Wright I, Reich PB (2003) Photosynthetic differences contribute to competitive advantage of evergreen angiosperm trees over evergreen conifers in productive habitats. New Phytol 160: 329–336. 10.1046/j.1469-8137.2003.00879.x 33832183

[ref53] Maherali H, Moura CF, Caldeira MC, Willson CJ, Jackson RB (2006) Functional coordination between leaf gas exchange and vulnerability to xylem cavitation in temperate forest trees. Plant Cell Environ 29: 571–583. 17080608 10.1111/j.1365-3040.2005.01433.x

[ref54] Maherali H, Pockman WT, Jackson RB (2004) Adaptive variation in the vulnerability of woody plants to xylem cavitation. Ecology 85: 2184–2199.

[ref55] Martínez-Vilalta J, Prat E, Oliveras I, Piñol J (2002) Xylem hydraulic properties of roots and stems of nine mediterranean woody species. Oecologia 133: 19–29. 24599365 10.1007/s00442-002-1009-2

[ref56] Martínez-Vilalta J, Sala A, Piñol J (2004) The hydraulic architecture of pinaceae–a review. Plant Ecol 171: 3–13.

[ref57] Martínez-Vilalta J, Mencuccini M, Álvarez X, Camacho J, Loepfe L, Piñol J (2012) Spatial distribution and packing of xylem conduits. Am J Bot 99: 1189–1196. 22733986 10.3732/ajb.1100384

[ref58] Mayr S, Hacke U, Schmid P, Schwienbacher F, Gruber A (2006) Frost drought in conifers at the alpine timberline: xylem dysfunction and adaptations. Ecology 87: 3175–3185. 17249241 10.1890/0012-9658(2006)87[3175:fdicat]2.0.co;2

[ref59] Newsome TM, Wolf C, Nimmo DG, Kopf RK, Ritchie EG, Smith FA, Ripple WJ (2020) Constraints on vertebrate range size predict extinction risk. Glob Ecol Biogeogr 29: 76–86.

[ref60] Niinemets Ü, Valladares F (2006) Tolerance to shade, drought, and waterlogging of temperate northern hemisphere trees and shrubs. Ecol Monogr 76: 521–547.

[ref61] Nobis MP, Traiser C, Roth-Nebelsick A (2012) Latitudinal variation in morphological traits of the genus pinus and its relation to environmental and phylogenetic signals. Plant Ecol Divers 5: 1–11.

[ref62] Oliveras I, Martínez-Vilalta J, Jimenez-Ortiz T, Lledó MJ, Escarré A, Piñol J (2003) Hydraulic properties of *Pinus halepensis*, *Pinus pinea* and *Tetraclinis articulata* in a dune ecosystem of Eastern Spain. Plant Ecol 169: 131.

[ref63] Pammenter NW, Vander Willigen C (1998) A mathematical and statistical analysis of the curves illustrating vulnerability of xylem to cavitation. Tree Physiol 18: 589–593. 12651346 10.1093/treephys/18.8-9.589

[ref64] Pérez-Harguindeguy N, Díaz S, Garnier E, Lavorel S, Poorter H, Jaureguiberry P, Bret-Harte MS, Cornwell WK, Craine JM (2013) Gurvich DE et al New handbook for standardised measurement of plant functional traits worldwide. *Aust J Bot* 61: 167–234.

[ref65] Pittermann J, Choat B, Jansen S, Stuart SA, Lynn L, Dawson TE (2010) The relationships between xylem safety and hydraulic efficiency in the cupressaceae: the evolution of pit membrane form and function. Plant Physiol 153: 1919–1931. 20551212 10.1104/pp.110.158824 PMC2923884

[ref66] Pittermann J, Sperry JS, Hacke UG, Wheeler JK, Sikkema EH (2005) Torus-margo pits help conifers compete with angiosperms. Science 310: 1924–1924. 16373568 10.1126/science.1120479

[ref67] Pittermann J, Stuart SA, Dawson TE, Moreau A (2012) Cenozoic climate change shaped the evolutionary ecophysiology of the Cupressaceae conifers. Proc Natl Acad Sci USA 109: 9647–9652. 22628565 10.1073/pnas.1114378109 PMC3386140

[ref68] Qian S, Yang Y, Tang CQ, Momohara A, Yi S, Ohsawa M (2016) Effective conservation measures are needed for wild Cathaya argyrophylla populations in China: insights from the population structure and regeneration characteristics. Forest Ecol Manag 361: 358–367.

[ref69] Ran J, Shen T, Wu H, Gong X, Wang X (2018) Phylogeny and evolutionary history of pinaceae updated by transcriptomic analysis. Mol Phylogenet Evol 129: 106–116. 30153503 10.1016/j.ympev.2018.08.011

[ref70] Ricklefs RE (2010) Evolutionary diversification, coevolution between populations and their antagonists, and the filling of niche space. Proc Natl Acad Sci USA 107: 1265–1272. 20080597 10.1073/pnas.0913626107 PMC2824412

[ref71] Scoffoni C, Chatelet DS, Pasquet-kok J, Rawls M, Donoghue MJ, Edwards EJ, Sack L (2016) Hydraulic basis for the evolution of photosynthetic productivity. Nat Plants 2: 1–8. 10.1038/nplants.2016.72 27255836

[ref72] Silvertown J (1982) Introduction to Plant Population, Longman Higher Education.

[ref73] Sperry J, Saliendra N (1994) Intra-and inter-plant variation in xylem cavitation in *Betula occidentalis*. Plant Cell Environ 17: 1233–1241.

[ref74] Sterck FJ, Martínez-Vilalta J, Mencuccini M, Cochard H, Gerrits P, Zweifel R, Herrero A, Korhonen JF, Llorens P, Nikinmaa E (2012) Understanding trait interactions and their impacts on growth in Scots pine branches across europe. Funct Ecol 26: 541–549.

[ref75] Taggart RE, Cross AT (2009) Global greenhouse to icehouse and back again: the origin and future of the boreal forest biome. Glob Planet Change 65: 115–121.

[ref76] Thompson RS, Anderson KH, Bartlein PJ (1999) Atlas of Relations Between Climatic Parameters and Distributions of Important Trees and Shrubs in North America. US Department of the Interior, US Geological Survey.

[ref77] Tyree MT, Sperry JS (1988) Do woody plants operate near the point of catastrophic xylem dysfunction caused by dynamic water stress?: Answers from a model. Plant Physiol 88: 574–580. 16666351 10.1104/pp.88.3.574 PMC1055627

[ref78] Vaario L-M, Xing S-T, Xie Z-Q, Lun Z-M, Sun X, Li YH (2006) In situ and in vitro colonization of cathaya argyrophylla (pinaceae) by ectomycorrhizal fungi. Mycorrhiza 16: 137–142. 16292663 10.1007/s00572-005-0026-5

[ref79] Vidaković M (1991) Conifers: Morphology and Variation, Grafičko Zavod Hrvatske.

[ref80] Wang H, Ge S (2006) Phylogeography of the endangered Cathaya argyrophylla (Pinaceae) inferred from sequence variation of mitochondrial and nuclear DNA. Mol Ecol 15: 4109–4122. 17054506 10.1111/j.1365-294X.2006.03086.x

[ref81] Wang M, Wang J, Zhang A, Zhang X, Sun S, Zhao C (2018) Functional traits related to environmental divergence in combination with phylogenetic relationship of picea species. Dendrobiology 80: 131–142.

[ref82] Webb CO, Ackerly DD, Kembel SW (2008) Phylocom: software for the analysis of phylogenetic community structure and trait evolution. Bioinformatics 24: 2098–2100. 18678590 10.1093/bioinformatics/btn358

[ref83] Wheeler JK, Sperry JS, Hacke UG, Hoang N (2005) Inter-vessel pitting and cavitation in woody Rosaceae and other vesselled plants: a basis for a safety versus efficiency trade-off in xylem transport. Plant Cell Environ 28: 800–812.

[ref84] Wiens JJ, Ackerly DD, Allen AP, Anacker BL, Buckley LB, Cornell HV, Damschen EI, Jonathan Davies T, Grytnes JA, Harrison SP (2010) Niche conservatism as an emerging principle in ecology and conservation biology. Ecol Lett 13: 1310–1324. 20649638 10.1111/j.1461-0248.2010.01515.x

[ref85] Wiens JJ, Graham CH (2005) Niche conservatism: integrating evolution, ecology, and conservation biology. Annu Rev Ecol Evol Syst 36: 519–539.

[ref86] Willson CJ, Jackson RB (2006) Xylem cavitation caused by drought and freezing stress in four co-occurring Juniperus species. Physiol Plant 127: 374–382.

[ref87] Willson CJ, Manos PS, Jackson RB (2008) Hydraulic traits are influenced by phylogenetic history in the drought-resistant, invasive genus Juniperus (Cupressaceae). Am J Bot 95: 299–314. 21632355 10.3732/ajb.95.3.299

[ref88] Wright IJ, Reich PB, Cornelissen JH, Falster DS, Garnier E, Hikosaka K, Lamont BB, Lee W, Oleksyn J, Osada N (2005) Assessing the generality of global leaf trait relationships. New Phytol 166: 485–496. 15819912 10.1111/j.1469-8137.2005.01349.x

[ref89] Xie Z, Chen W (1999a) The endangering causes and preserving strategies for cathaya argyrophylla, a plant endemic to China. Acta Ecologica Sinica 23: 1–7.

[ref90] Xie Z, Chen W, Lu P, Hu D (1999b) The demography and age structure of the endangered plant population of Cathaya argyrophylla. Acta Ecologica Sinica 19: 523–528.

[ref91] Xie Z, Li Q (2000) Seed characteristics of endangered plant Cathaya argyrophylla. Acta Phytoecol Sinica 24: 82–86.

[ref92] Xiong D, Nadal M (2019) Linking water relations and hydraulics with photosynthesis. Plant J 101: 800–815. 31677190 10.1111/tpj.14595

[ref93] Yamori W, Noguchi K, Terashima I (2005) Temperature acclimation of photosynthesis in spinach leaves: analyses of photosynthetic components and temperature dependencies of photosynthetic partial reactions. Plant Cell Environ 28: 536–547.

[ref94] Ying J, Ma C, Li L, Zhang Z, Zhang W (1983) Studies on the Cathaya communities. Acta Bot Sinica 25: 157–169.

[ref95] Zanne AE, Tank DC, Cornwell WK, Eastman JM, Smith SA, FitzJohn RG, McGlinn DJ, O’Meara BC, Moles AT, Reich PB (2014) Three keys to the radiation of angiosperms into freezing environments. Nature 506: 89–92. 24362564 10.1038/nature12872

[ref96] Zhang W, Fan D, Xie Z, Jiang X (2005) The seasonal photosynthetic responses of seedlings of the endangered plant Cathaya argyrophylla to different growth light environments. Biodiversity Sci 13: 387–397.

[ref97] Zimmermann M (1983) Xylem Structure and the Ascent of Sap. Springer-Verlag, Berlin.

